# Permanent Maxillary Canine Agenesis: A Rare Case Report

**DOI:** 10.5005/jp-journals-10005-1322

**Published:** 2015-09-11

**Authors:** Halaswamy V Kambalimath, Somya Jain, Raju Umaji Patil, Alexander Asokan, Deepashri Kambalimath

**Affiliations:** Professor and Head, Department of Pedodontics and Preventive Dentistry Rishiraj College of Dental Science and Research Centre Bhopal, Madhya Pradesh, India; Postgraduate Student, Department of Pedodontics and Preventive Dentistry Rishiraj College of Dental Science and Research Centre Bhopal, Madhya Pradesh, India; Reader, Department of Pedodontics and Preventive Dentistry Rishiraj College of Dental Science and Research Centre Bhopal, Madhya Pradesh, India; Senior Lecturer, Department of Pedodontics and Preventive Dentistry Rishiraj College of Dental Science and Research Centre Bhopal, Madhya Pradesh, India; Senior Lecturer, Department of Oral and Maxillofacial Surgery, Rishiraj College of Dental Science and Research Centre, Bhopal, Madhya Pradesh, India

**Keywords:** Agenesis, Bilateral, Maxillary canines, Congenitally missing.

## Abstract

Congenitally missing teeth (CMT) are among one of the commonly known dental anomalies. The most frequently missing teeth in the permanent dentition, excluding the third molars, are mandibular second premolars and maxillary lateral incisors. Exclusive agenesis of both maxillary canines is an extremely rare occurrence and only a few cases have been reported. Previous studies showed that the prevalence of maxillary canine agenesis varies between 0.07 and 0.13%. In recent studies on Indian population, no cases of maxillary canine agenesis have been documented. This paper reports a case of non-syndromic bilateral agenesis of permanent maxillary canines, along with agenesis of both mandibular central incisors in a healthy 13-year-old Indian female patient; and a brief literature review on prevalence, etiology and treatment modalities of the condition.

**How to cite this article:** Kambalimath HV, Jain S, Patil RU, Asokan A, Kambalimath D. Permanent Maxillary Canine Agenesis: A Rare Case Report. Int J Clin Pediatr Dent 2015; 8(3):242-246.

## INTRODUCTION

Congenitally missing teeth (CMT) are among one of the commonly known dental anomalies;^1^ which can be defined as developmental absence of teeth, except the third molars, either in primary or permanent dentition.^2^ It has also been termed as teeth aplasia, teeth agenesis, and lack of teeth.^3^ The anomaly CMT can be classified in a number of ways. Firstly, on the basis of number of missing teeth, it is termed as ‘hypodontia’ (< 6 missing teeth), oligodontia’ (≥ 6 missing teeth) and ‘anodontia’ (complete absence of teeth).^4^ Secondly, according to the severity of condition, it can be classified as ‘mild to moderate hypodontia’ (2-5 teeth absence), ‘severe hypodontia’ (≥ 6 teeth absence) and ‘oligodontia’ (multiple teeth absence in relation to systemic disorders).^5^ It can further be divided into ‘syndromic’ or ‘non-syndromic’ forms, and can appear either sporadically or as an inherited condition.^6^

Large variations ranging from 0.3 to 34.3% in the prevalence rate of CMT based on ethnicity and continents have been reported in cases of permanent dentition.^7,8^ On the contrary, tooth agenesis is rare in primary dentition, with a prevalence rate ranging between 0.1 and 0.9%.^2,9^ Occurrence of CMT in permanent dentition has been considered to be the most common reason for primary tooth retention.^10^

Tooth agenesis in relation to gender has demonstrated a relatively higher incidence in females as compared to males.^2,11,12^ Literature also reveals the difference in the occurrence rate of CMT in the anterior and posterior region, with a greater predilection for anterior region.^13,14^ Also, differences have been reported in the prevalence of CMT in maxilla and mandible, as well as in unilateral and bilateral occurrence. Some studies reveal tooth agenesis to be more common in maxilla which is in contrast to other studies.^15-17^ Unilateral agenesis occurs more frequently as compared to bilateral agenesis, with the exception of maxillary lateral incisors.^11^

The tooth most commonly found to be missing is the third molar, followed by mandibular second premolars (41%), maxillary lateral incisors (23%), maxillary second premolars (21%), and the mandibular incisors (6%).^2^ The occurrence of tooth agenesis has thus been subdivided into three categories (Table 1).^11^

Congenital absence of permanent canines has been reported as a rare occurrence, though several studies have reported hypodontia affecting maxillary canine.^12^ It may occur as part of a syndrome or as a non-syndromic form.^18^ Previous reports on congenital absence of permanent maxillary canines have been tabulated in Table 2.^8,11,15,16,19-28^ Different studies have reported greater frequency of occurrence of agenesis in the maxillary region along with higher female predilection and more chances of unilateral agenesis.^24,27,29^ The association of canine agenesis with other dental abnormalities like microdontia, agenesis of other teeth, supernumerary teeth, malocclusion and retained primary teeth have been shown.^27^ Recently, in the studies done on Indian population by Guttal et al in 2010, Gupta et al in 2011 and Shetty et al in 2012, no cases of maxillary canine agenesis have been reported.^29-31^ Multifactorial etiology of CMT, which combines genetic, epigenetic and environmental factors, is noted.^2,12^ The genetic or the familial inheritance has been attributed as a more significant etiological factor. Autosomal dominant (AD), autosomal recessive (AR) and X-linked recessive pattern of inheritance have been associated with tooth agenesis; with AD pattern being the most prominent.^18,32^ Some of the regulatory homeobox genes—MAX1, PAX9, EDA and AXIN2 have been found in association with tooth agenesis.^18^ Environmental factors like tooth bud infection, trauma, nutritional disturbances during pregnancy or infancy, smoking during pregnancy, maternal medications, irradiation at an early stage and somatic diseases (syphilis, scarlet fever and rickets) are also associated with tooth agenesis.^2,12^

The association of tooth agenesis with other syndromes as well as other dental anomalies have also been reported in the literature. Congenitally missing teeth have been frequently reported in cases of oral and facial clefts, Rieger syndrome, Down syndrome, Witkop syndrome, Book syndrome, hemifacial microsomia and many others.^2,12^ Tooth agenesis has also been shown to accompany other conditions, such as microdontia, palatal impaction of canines, taurodontism, tooth transposition and rotation, ectopic eruption, retained primary teeth and alveolar bone hypoplasia.^2,12,33^

The aim of this article is to present a rare case report of congenitally missing bilateral permanent maxillary canines along with agenesis of permanent central incisors in the mandibular region.

**Table 1:** **Table 1:** Sequence of most to least affected teeth, divided into three categories

*Category*		*Prevalence (%)*		*Sequence*	
Common		1.5 to 3.1		Mand. 2nd PM > Max. LI > Max. 2nd PM	
Less common		0.1 to 0.3		Mand. CI > Mand LI and Max. 1st PM > Max. Canine and Mand. 2nd Molar	
Rare		0.01 to 0.04		Max. 2nd Molar and Mand. 1st Molar > Mand. Canine > Mand. 1st molar and Max. CI	

PM: Premolar; LI: lateral incisors; CI: canine incisors

**Table 2:** **Table 2:** Previous reports on congenital absence of permanent maxillary canines

*Sl. no.*		*Years*		*Authors*		*Prevalence*	
1.		1937		Dolder E		0.06	
2.		1966		Rose		0.12	
3.		1977		Bergstorm		0.23	
4.		1987		Davis		0.45	
5.		2000		Hokari et al		0.26	
6.		2004		Fukuta et al		0.18	
7.		2004		Polder’s meta-analysis		0.3	
8.		2005		Fekonja		2.1	
9.		2007		Sismana et al		0.37	
10.		2008		Harris and Clark		0.4	
11.		2008		Goya et al		0.5	
12.		2009		Roza		0.27	
13.		2012		Sheikhi et al		1.98 to 2.20	
14.		2012		Shetty et al		0	

## CASE REPORT

**Fig. 1:**
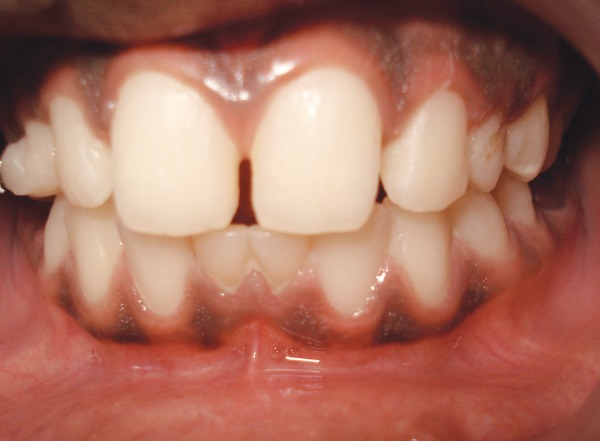
Intraoral photograph showing retained deciduous maxillary right and left canines and mandibular both right and left central incisors

**Fig. 2:**
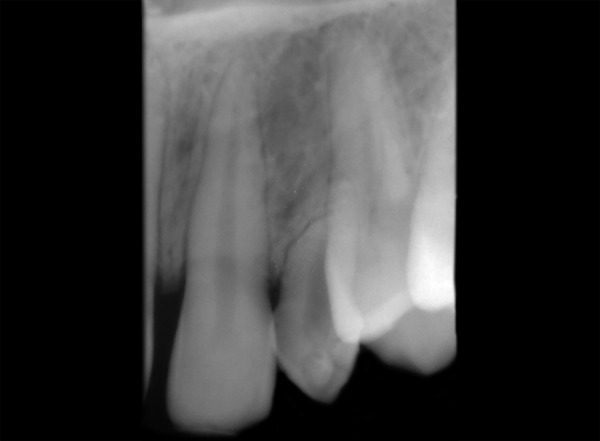
Periapical radiograph showing absence of right maxillary permanent canine

**Fig. 3:**
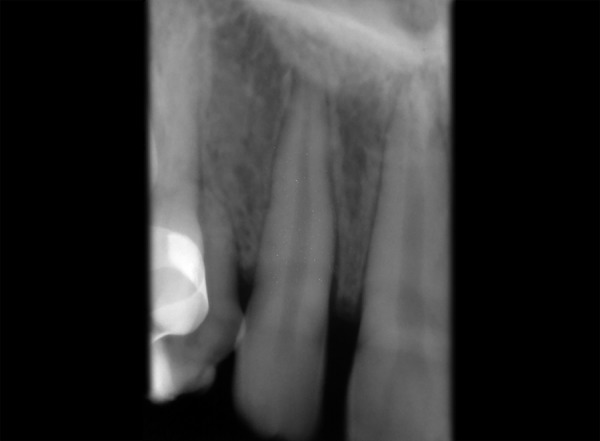
Periapical radiograph showing absence of left maxillary permanent canine

**Fig. 4:**
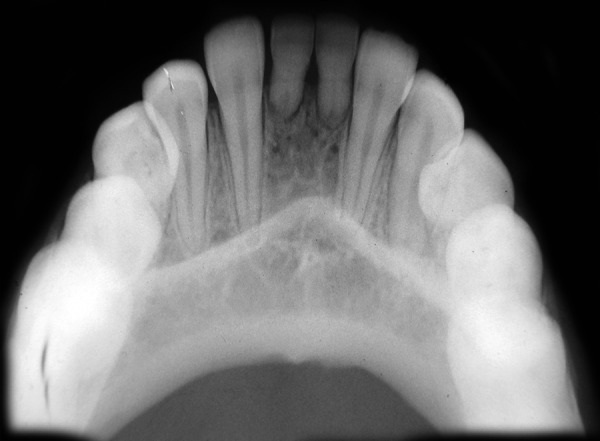
Mandibular occlusal radiograph showing bilateral absence of permanent mandibular central incisors

**Fig. 5:**
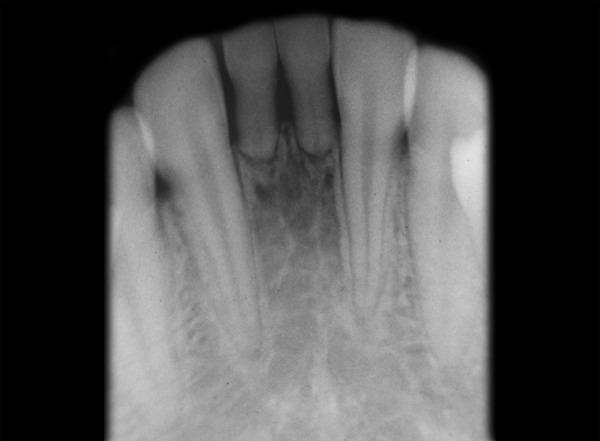
Periapical radiograph showing agenesis of both mandibular central incisors

**Fig. 6:**
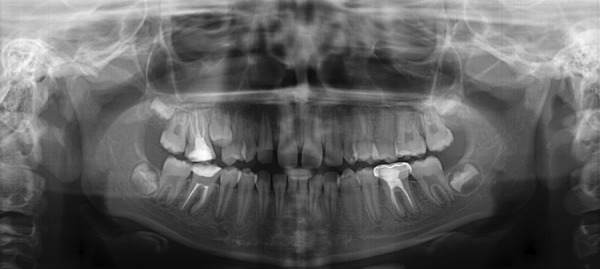
Panaromic view of bilateral agenesis of maxillary canines and mandibular central incisors

A 13-year-old healthy female patient reported to the department of pedodontics and preventive dentistry, Rishi Raj College of Dental Science and Research Centre, with the complaint of pain in the right lower back tooth region. Clinical examination revealed retained deciduous maxillary canines on both sides along with both lower central incisors (Fig. 1). No mobility in deciduous teeth was found. The prenatal, natal and post-natal history was not significant. Also, there was no history related to trauma or infections in the anterior region. Family history and medical history were also not significant. Suspecting the congenital absence of both permanent maxillary canines and lower central incisors, various radiographs were taken to confirm the provisional diagnosis. Radio-graphic examination revealed congenital absence of bilateral maxillary canines as well as central incisors in the mandibular region (Figs 2 to 6). Also, insufficient space for the eruption of right second premolar was found both clinically and radiographically. Patient had been informed regarding the absence of teeth.

## DISCUSSION

Permanent maxillary canines are known to be one of the most variably positioned teeth in the oral cavity with palatal or facial displacement or ectopically eruption from the dental arch. Congenital canine agenesis is a rare condition.^34^

The different rates of prevalence of tooth agenesis on the basis of the tooth type correlates with the Butler’s field theory for mammalian teeth. The theory states that the tooth that is situated most mesially is the most stable tooth in each morphological class, and as canine is the only representative element in its developmental field, it is considered to be the most stable and rarely missing tooth.^35^ This theory was applied for the human dentition by Dahlberg in 1945. According to his concept, there is presence of a ‘key tooth’ (stable genetically) mesially in each developmental field, while distal end of the field has presence of teeth which is least stable.^36^ This concept was taken as basis by Bailit to explain the variation in tooth agenesis in the permanent dentition. He classified teeth into two groups—stable and unstable; the upper canines are considered stable along with the upper central incisors, the first premolars, and the first molars, and thus their agenesis was considered to be rare.^37^ Tooth agenesis has been known to have detrimental effects on one’s esthetics and can impair masticatory ability, speech development and most importantly, can emotionally upset an individual during adolescent years.^33^ It also results in dental malpositioning, periodontal damage, lack of development of maxillary and mandibular bone height.^38^

Haselden et al reported a longer survival rate of primary canines without permanent successors.^39^ This over-retention of primary canines can be functionally useful in cases with severe hypodontia, as retention of primary canines may impede the resorption of the alveolar bone, and thus can help in preservation of bone volume and may be favorable for consideration of implants as a treatment alternative.^40^ Significant growth changes takes place in the upper part of the mandibular symphysis during childhood and puberty, and is related with continuous eruption of lower incisors. Therefore, agenesis of lower incisors may have an influential impact on the growth of mandibular symphyseal region.^41^

Congenitally missing maxillary permanent canines pose a particular challenge in treatment planning. Factors to be considered include—the condition of the primary predecessor, the number of missing teeth, the overall alignment and occlusion, and most importantly, the patient’s and/or parents’ preferences.^40^

Treatment options may include timely extraction of the primary predecessors to facilitate spontaneous space closure with or without further orthodontic alignment, followed by lateral incisor and first premolar coronoplasty, or to keep the primary canines and replace them with a suitable restoration when they are lost. An advantage of retaining the primary predecessor is that, with the growing use of implants, alveolar resorption may be avoided until the late teens, providing the maximum potential for implant placement without the need for bone grafting. Each patient has to be assessed individually to decide the most suitable treatment plan. Referral to an orthodontist and/or prosthodontist for definitive treatment will be needed for most cases.^34,42^

In the present case, the main aim was to preserve the retained deciduous maxillary canines and mandibular central incisors, as far as possible. It was decided to keep a regular follow-up every 3 months, and as the root resorption of the deciduous teeth have already begun, a future referral to an orthodontist/prosthodontist will be needed.

## CONCLUSION

The occurrence of this trait demands for a multidisciplinary team management with the aim to maintain the existing dentition, improve esthetics and speech, allow proper mastication, and promote the child’s emotional and psychological well-being. The role of pediatric dentist is to: manage the child’s behavior, maintain good oral hygiene, manage malocclusion; and provide intermediate restorations like removable or fixed partial dentures and resin retained bridges.
